# MicroRNA-152 Regulates DNA Methyltransferase 1 and Is Involved in the Development and Lactation of Mammary Glands in Dairy Cows

**DOI:** 10.1371/journal.pone.0101358

**Published:** 2014-07-02

**Authors:** Jie Wang, Yanjie Bian, Zhuoran Wang, Dan Li, Chunmei Wang, Qingzhang Li, Xuejun Gao

**Affiliations:** Key Laboratory of Dairy Science of Education Ministry, Northeast Agricultural University, Harbin, Heilongjiang, China; Florida State University, United States of America

## Abstract

MicroRNAs (miRNAs) are a class of small non-coding, endogenous regulatory RNAs that function by controlling gene expression at the post-transcriptional level. Using small RNA sequencing and qRT-PCR techniques, we found that the expression of miR-152 was significantly increased during lactation in the mammary glands of dairy cows producing high quality milk compared with that in cows producing low quality milk. Furthermore, DNA methyltransferase 1 (DNMT1), which is a target of miR-152, was inversely correlated with the expression levels of miR-152 in the mammary glands of dairy cows. Dairy cow mammary epithelial cells (DCMECs) were used as in vitro cell models to study the function of miR-152. The forced expression of miR-152 in DCMECs resulted in a marked reduction of DNMT1 at both mRNA and protein levels. This in turn led to a decrease in global DNA methylation and increased the expression of two lactation-related genes, serine/threonine protein kinase Akt (*Akt*) and peroxisome proliferator-activated receptor gamma (*Pparγ*). In contrast, inhibition of miR-152 showed the opposite results. By using an electronic Coulter counter (CASY-TT) and flow cytometer, we discovered that miR-152 enhanced the viability and multiplication capacity of DCMECs. In conclusion, miR-152 plays an important role in the development and lactation processes in the mammary glands of dairy cows. Our data provide insights into dairy cow mammary gland development and lactation.

## Introduction

MicroRNAs (miRNAs) are endogenous, non-coding, functional RNAs. Mature miRNA is a double-stranded RNA that contains approximately 22 nucleotides long generated from pre-miRNAs by the mediator of the RNAse III family member called Dicer [1]. The miRNAs are post-transcriptional regulators that bind by complementary base pairing to sequences in the 3′ untranslated regions (3′-UTRs) of target mRNAs by translational repression or degradation of the transcript [2], [3]. Abundant evidence has shown that miRNAs play an important role in various physiological process [4], such as cell differentiation, cell growth, and cell death [5].

MiR-152 is one of the three members of the miR-148/152 family, which contain miR-148a, miR-148b and miR-152. Recent studies have demonstrated that the aberrant expression of miRNAs in mammary glands may have crucial effects on mammary gland development and lactation [6]–[9]. We found that the expression of miR-152 differed in cow mammary gland tissues during the various lactation periods using small RNA sequencing and qRT-PCR. However, the involvement of miR-152 in the mammary gland at the molecular level is poorly understood. Therfore, it was proposed that miR-152 could regulate mammary gland development and lactation at the post-transcriptional level in Holstein dairy cows.

DNA methylation is an important epigenetic modification that participates in the regulation of gene expression. DNA methylation is catalyzed by enzymes in the DNA methyltransferase (DNMT) family, which have three members: DNMT1, DNMT3a and DNMT3b. DNMT3a and DNMT3b are responsible for de novo methylation of unmodified DNA, whereas the enzymatic activity of DNMT1 affects the maintenance of DNA methylation during DNA replication [10]. Each enzyme catalyzes the transformation of a methyl group from S-adenosyl-L-methionine to a cytosine base at the carbon 5 position (5 meC) in DNA. Methylation is often found in CpG and CpHpG (H = A, T, C) sequences. When it is located at gene promoters, methylation usually represses the expression of genes [11]. It had been reported that the methylation status of the STAT5 promoter region acutely affects α-casein expression in bovine mammary gland tissue [12], [13]. In addition, DNMT1 is necessary and sufficient to maintain global methylation and aberrant CpG island methylation in human cancer cells [14]. Both accumulated evidence and a previous study from our group indicate that miRNAs are involved in global DNA hypomethylation through their targeting of DNMTs in 3′ UTRs [15]–[20]. Therefore, in the present study, we tested whether miR-152 regulates DNMT1, which in turn influences lactation-related genes in dairy cow mammary epithelial cells (DCMECs).

## Materials and Methods

### Animals and mammary gland samples

In this study, six healthy multiparous Holstein cows in mid-lactation period (100 days postpartum) averaging 609±9.08 kg (mean±s.e.) live weight and a parity of 3.1±0.19 over three generations were obtained from the Holstein Cattle Association of Australia. The cows were divided into two groups according to the quality of their milk: one group was in a lactation period with high milk quality (milk yield 30.8±0.76 kg/d, milk protein >3.0%, milk fat >3.5%) and another group was in a lactation period with low milk quality (milk yield 30.6±0.78 kg/d, milk protein <3.0%, milk fat <3.5%) (n = 3 animals per group). Once a day, both groups were provided a standard mixed ration that comprised 30% roughage and 70% concentrate. Cows were slaughtered by exsanguination, and mammary gland tissue was aseptically excised 5 cm from the base of the healthy nipple and 3 cm from the half line that divides the core of the secretory gland tissue. After removing the connective tissue, the remaining tissue was cut into small blocks with a thickness of 1 cm. Mammary gland tissue samples were frozen immediately in liquid nitrogen and stored at −80°C for further analysis. All animal experiments were approved by the Institutional Animal Care and Use Ethics Committee of Northeast Agricultural University and conducted in accordance with the Guidelines for Experimental Animals from the Ministry of Science and Technology (Beijing, China).

### Small RNA sequencing

Total RNA was extracted from the mammary gland tissues of the two groups (n = 3 animals per group) using Trizol reagent (Invitrogen, Carlsbad, CA, USA) according to the manufacturer's instructions. Subsequently, the RNA samples were sent to Beijing Genomics Institute (Shenzhen, Guangdong, China) for constructing small RNA libraries and sequencing using a Genome Analyzer (Illumina, San Diego, USA). The sequenced short reads data have been deposited to the Short Archive section of GEO at NCBI under accession numbers GSE57991. The sequencing data were first filtered into mRNA using Rfam 10.1 and Genebank databases and then mapped to miRbase 18. The mapped data were used to identify significant differences in the expression of the miRNAs.

### Cell culture and transfection

The DCMECs were obtained from the Key Laboratory of Dairy Science of Education Ministry (Northeast Agricultural University, Harbin, China). Cytokeratin-18 was identified with immunohistochemistry using a laser scanning confocal microscope (Leica TCS SP2 AOBS, Germany) (Figure S1) [21]. Dulbecco's modified Eagle's medium-F12 (Gibco, Grand Island, NY, USA) containing 10% fetal bovine serum (FBS) (Invitrogen), 100 U/ml penicillin and 100 µg/ml streptomycin was used for routine maintenance of the DCMECs. The DCMECs were maintained in a culture bottle with medium at 37°C and an atmosphere of 5% CO_2_.

The HEK-293 cells were purchased from the American Type Culture Collection. The cells were maintained in Dulbecco's modified Eagle's medium-F12 (Gibco) containing 10% FBS (Invitrogen), and antibiotics (100 U/ml penicillin and 100 µg/ml streptomycin) in a humidified incubator at 37°C with 5% CO_2_.

The DCMECs were transfected with miR-152 mimics (miR-152), miR-152 inhibitors (Anti-152) and negative controls (miR-NC, Anti-NC) (GenePharma, Shanghai, China) using siRNA-Mate (GenePharma) according to the manufacturer's instructions.

### Drug treatment with 5-aza-2′-deoxycytidine

DCMECs were grown in medium containing a final concentration of 0.1 µM 5-aza-2′-deoxycytidine (5-Aza) (Sigma, Oakville, ON, Canada) for 48 h to block both de novo and maintenance DNMT pathways according to previous studies [22], [23]. Total RNA and cellular proteins were extracted and subjected to different analyses.

### Total RNA preparation and qRT-PCR analysis

Total RNA was extracted using Trizol reagent (Invitrogen) according to the manufacturer's instructions. The quantity and purity of RNA were verified by measuring the absorbance at 260 nm (A260) and A280. Total RNA integrity was verified by an optical density (OD) ratio of OD260/OD280 greater than 1.8. For each sample, 1 µg of total RNA was used to synthesize the first cDNA strand with the PrimeScript RT Reagent Kit (TaKaRa, Tokyo, Japan).

The TaqMan MicroRNA Assay Kit (GenePharma) was used to detected the expression of mature miR-152 and the internal control 5S gene. The specific primers for miR-152 and 5S were designed by GenePharma. PCR reactions were performed using the ABI PRISM 7300 Real-Time PCR System (Applied Biosystems, Foster City, CA, USA). The reaction was started with 3 min of incubation at 95°C followed by 40 cycles of 95°C for 12 s and 62°C for 40 s.

SYBR Premix Ex TaqTM II (TaKaRa) was used to measure the expression of DNMT1 and other genes of interest. β-actin served as a normalization control. The qRT-PCR analysis was conducted by denaturing at 95°C for 10 min, followed by 40 cycles of 95°C for 12 s and 62°C for 40 s. The primers used for qRT-PCR are listed in Table 1. All reactions were performed in triplicate. The relative levels of mRNA expression were calculated using the comparative 2^−ΔΔCt^ method [24].

**Table 1:** Genes and primers for fluorescence-based quantitative real-time PCR.

Gene name	Primer(5′-3′)	Amplicon size
DNMT1	F:TTGGTCTTGGACACTACGGCTAT	273 bp
	R:TAGCAAACGGAGACCAGAAGAA	
PPARγ	F:TCAAAGTGGAGCCTGTATC	138 bp
	R:CATAGTGGAACCCTGACG	
AKT	F:TAAAGAAGGAGGTCATCGTGG	181 bp
	R:CGGGACAGGTGGAAGAAAA	
β-actin	F:AAGGACCTCTACGCCAACACG	249 bp
	R:TTTGCGGTGGACGATGGAG	

### Immunohistochemistry

Frozen sections harvested from cows in lactation periods with high and low milk qualities were fixed in acetone at 4°C for 10 min. The tissue sections on slides were washed with phosphate buffered saline (PBS) containing 5% Tween-20 (PBST) and incubated with 5% BSA at 37°C for 60 min. The slides were then incubated with anti-DNMT1 antibody (1∶200, Abcam, Cambridge, MA, USA) in a humid chamber at 4°C overnight, rinsed three times with PBST and incubated for 60 min with TRITC-conjugated goat anti-rabbit IgG (1∶200, Zhongshan-Bio, Beijing, China). The slides were then washed three times with PBST and incubated in 1 µg/ml propidium iodide (PI) (Roche, Florence, Carolina, USA) for 10 min. Finally, 100 µL Antifade Mounting Medium (Beyotime, China) was added. We viewed the slides under a laser scanning confocal microscope (Leica, Germany). Image-Pro Plus (IPP) 6.0 software was used for detecting the mean density of the DNMT1 expression. Three sections from each group were used to quantify the DNMT1 protein.

### Vector construction

For luciferase reporter measurements, a 132-bp segment of DNMT1 from the 3′ UTR that included the putative miR-152 binding site was amplified by PCR. The following primers obtained from GenePharma were used to yield specific fragments: DNMT1 3′ UTR sense, 5′-AAGCTTATGTCAGCCAAGGCCACAA-3′; DNMT1 3′ UTR antisense 5′-ACTAGTCTATCACCCATGTTTCTGCC-3′. Underlined sequences show the endonuclease restriction sites. After digestion of the PCR products by HindIII and SpeI, The amplified 3′ UTR of DNMT1 was inserted into the HindIII and SpeI sites of the miRNA expression reporter vector system pMIR-Report (Ambion, Austin, TX, USA) downstream of the luciferase gene, generating pMIR-Report-DNMT1, which was used to perform the luciferase assay.

### Luciferase Reporter Assay

The pMIR-Report-DNMT1 was transfected into DCMECs and HEK-293 cells in 24-well plates. In each well, 0.08 µg of Renilla luciferase and phRL-TK vector (Promega, Madison, WI, USA) were cotransfected for normalizing the transfection efficiency. The firefly luciferase reporter vector (0.4 µg) together with 100 nM miR-152 or miR-NC were cotransfected into the DCMECs and HEK-293 cells using Lipofectamine 2000 (Invitrogen) according to the manufacturer's protocol. After 48 h, the cells were collected and analyzed using the Dual-Luciferase Reporter Assay System (Promega) according to the manufacturer's protocols. The experiments were performed in triplicate.

### Western blot analysis

Protein samples (50 µg) were solubilized in 2×SDS gel-loading buffer. The proteins were separated using 10% SDS-PAGE and electrotransferred to a PVDF membrane (Millipore, Billerica, MA, USA). The membranes were blocked in TBST buffer (TBS plus 0.1% Tween-20) with 5% w/v skimmed milk and incubated with primary antibodies (rabbit polyclonal antibodies) to PPARγ, DNMT1 (Abcam), Akt, phospho-Akt (Ser473) (Cell Signaling Technology, Beverly, MA, USA) and mouse polyclonal antibodies to β-actin (Santa Cruz Biotechnology Inc., California, USA). This was followed by an incubation with a specific HRP-conjugated secondary antibody (1∶2000, Zhongshan-Bio). Super ECL plus (ApplyGEN, Beijing, China) was used to measure protein bands. β-actin was used as a reference protein, and the results were analyzed with Bandscan version 4.3 software.

### Global DNA methylation (GDM) and DNMT activity studies

Genomic DNA was isolated from the DCMECs using a genomic DNA extraction kit (TaKaRa) according to the manufacturer's instructions. The levels of global DNA methylation in individual samples after transfection with miR-152, anti-miR-152 and their respective controls were determined using a 5-mC DNA ELISA Kit (ZYMO, Irvine, CA, USA)according to the manufacturer's protocol.

After the transfection as described above, the DCMECs were harvested, and the nuclear proteins were isolated with NE-PER Nuclear and Cytoplasmic Extraction (Thermo, Waltham, MA, USA). The protein extracts were quantified with a BCA Protein Assay Kit (Beyotime). The activity of the DNMT enzyme was determined using an EpiQuik DNA Methyltransferase Assay Kit (Epigentek, Farmingdale, NY, USA) according to the manufacturer's instructions.

### Detection of β-casein, triglyceride and lactose secretion

After transfection of the DCMECs as described above, the supernatant from the cultures was collected and the levels of the secreted β-casein, triglyceride and lactose were determined using the ELISA Kit for β-casein (CSN2) (New England Biolabs Inc., Beverly, MA, USA), Triglyceride (TG) GPO-POD Assay Kit (ApplyGEN) and Lactose & D-galactose (Rapid) Assay Kit (Megazyme, Bray, Ireland) according to the manufacturers' instructions. All experiments were performed in triplicate.

### Cell viability assay

DCMECs were transfected with miR-152, Anti-152, or their respective controls and then were incubated in microplates at 37°C with 5% CO_2_ for 0 h, 24 h, 48 h and 72 h. The cell viability at each time point was determined with a CASY-TT Analyser System (Schärfe System GmbH, Reutlingen, Germany) according to the manufacturer's instructions. After calibration with dead and vital DCMECs cells, cursor positions were set as 11.75 to 50.00 µm (evaluation cursor) and 7.63 to 50.00 µm (standardization cursor). After trypsinization, aliquots (100 µL) of the cells were diluted with the CASY electrolyte solution (1∶100) and analyzed in triplicate using the CASY-TT. All experiments were performed in triplicate.

### Cell cycle analysis

Forty-eight hours after the transfection (as described above), the DCMECs were harvested by centrifugation, washed in PBS and fixed in 70% alcohol at 4°C overnight. After being resuspended twice with PBS, DNA was stained with PI (50 µg/ml) in TritonX-100 (2 µl/ml) for 20 min at room temperature in the dark. The cells were rinsed in 500 µl of PBS and introduced into a Cytomics FC500 flow cytometer. The results were analyzed with ModFit LT 3.2 software (Verity Software House, USA). The flow cytometric analysis was repeated three times.

### Statistical analysis

Data are presented as means ± standard deviation from at least three separate experiments. Statistical analyses were performed using SPSS 17.0 software (Chicago, IL, USA). Tukey's post hoc tests were used for comparison between groups. Results with P<0.05 were considered statistically significant.

## Results

### MiR-152 is inversely correlated with DNMT1 in mammary gland tissues of cows with different milk qualities

**Figure 1:**
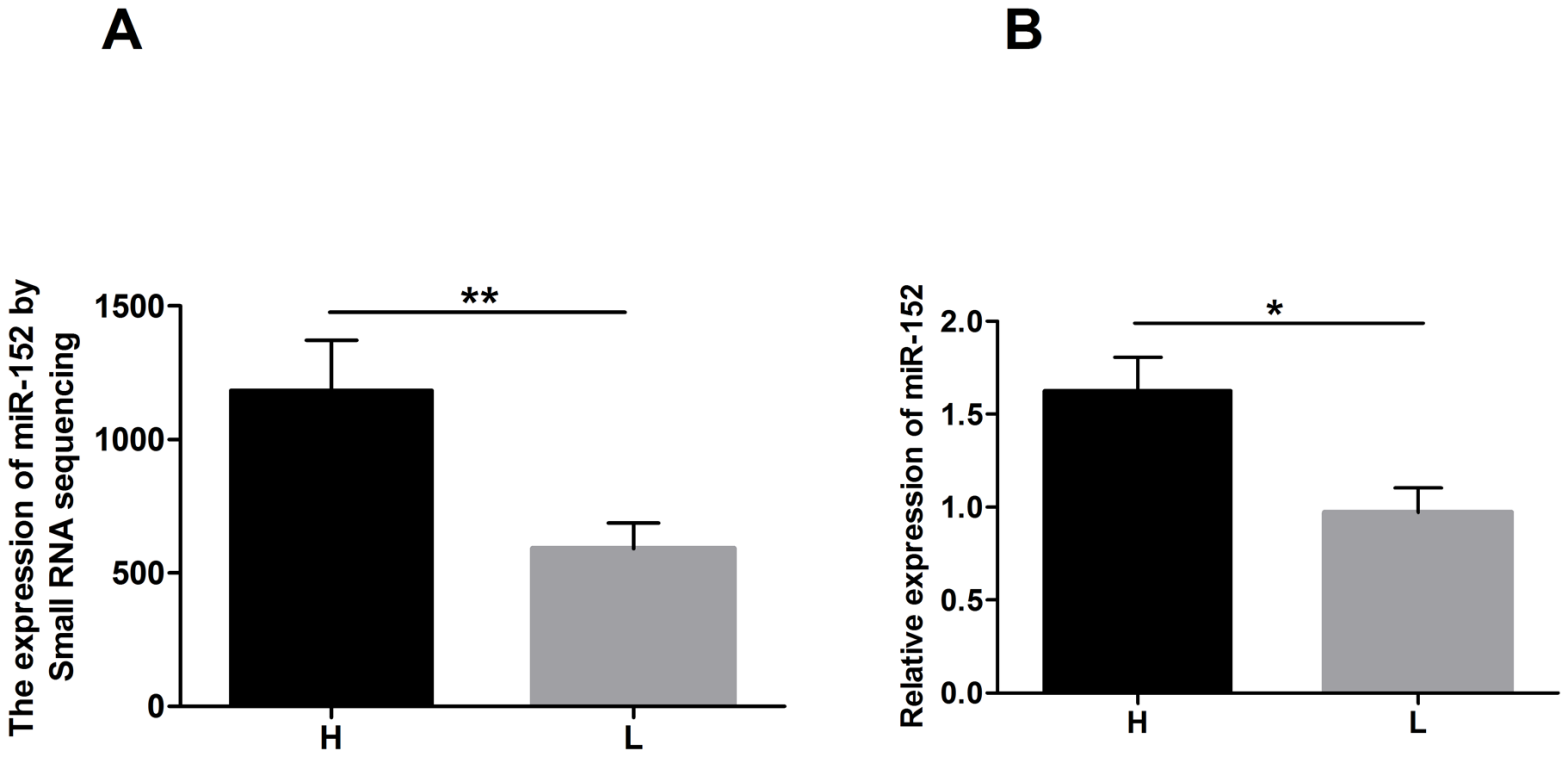
The expression of miR-152 in mammary glands from dairy cows producing different milk qualities. A: Small RNA sequencing results for miR-152; B: The mRNA expression levels of miR-152 determined by qRT-PCR. H: mammary gland tissues from cows producing high quality milk; L: mammary gland tissues from cows producing low quality milk. The relative expression of miR-152 was determined in two groups (n = 3 animals per group). QRT-PCR was repeated three times for *miR-152* detection. *miR-152* is up-regulated in mammary gland tissues of cows producing high quality milk compared with those producing low quality milk. *P<0.05, **P<0.01.

We assessed the expression of miR-152 in mammary gland tissues from cows using both small RNA sequencing and qRT-PCR techniques. Using small RNA sequencing, we found that the level of miR-152 was significantly up-regulated in the mammary gland tissues of cows producing high quality milk (H) compared with that in cows producing low quality milk (L) as shown in Figure 1A (*P*<0.01). We also observed that the expression of miR-152 was markedly higher in the mammary gland tissues of cows producing H compared with those producing L using qRT-PCR, validating the small RNA sequencing results (Figure 1B, *P*<0.05).

**Figure 2:**
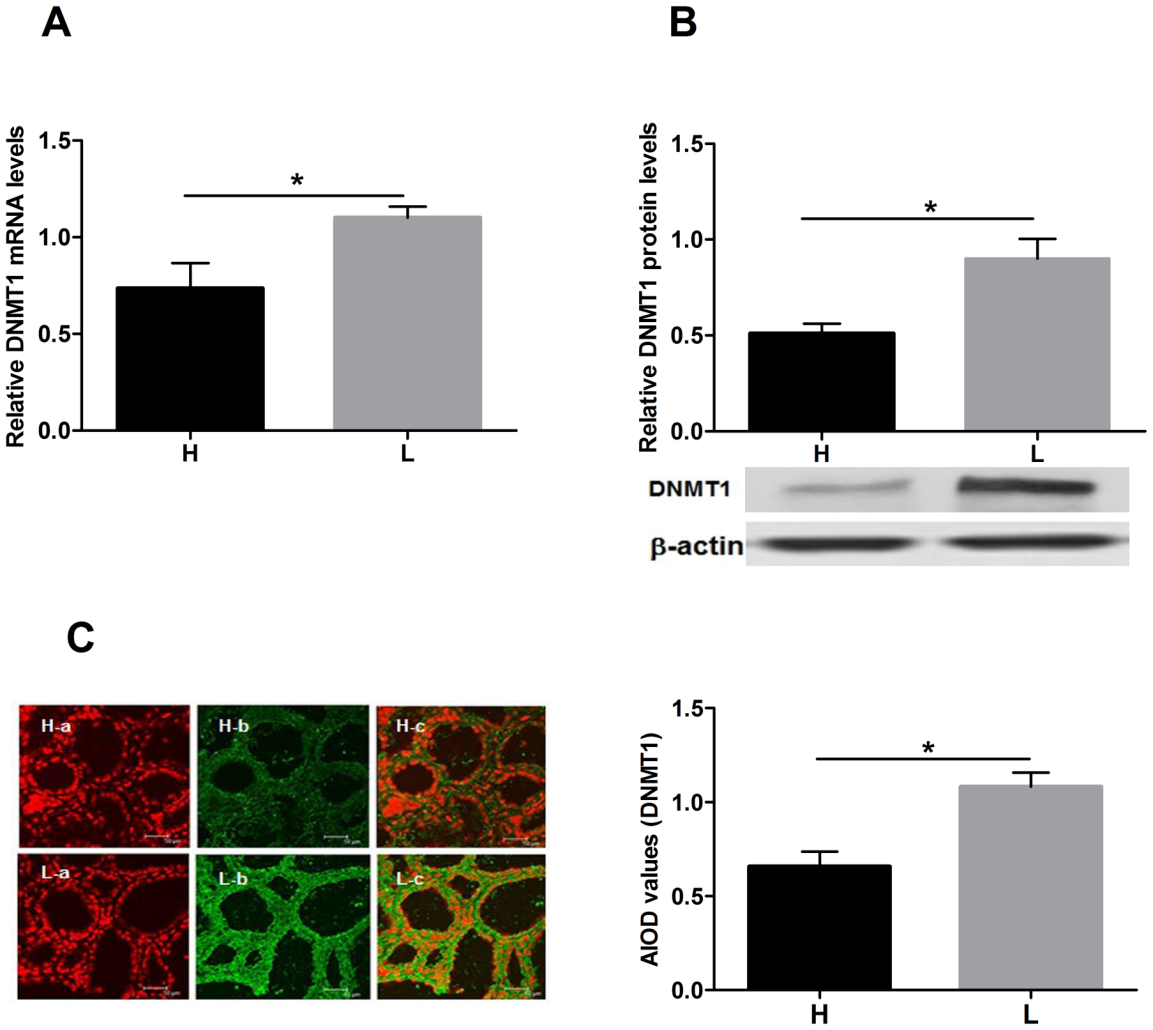
DNMT1 mRNA and protein expression in mammary gland tissues from dairy cows. A: The mRNA expression levels of *Dnmt1* determined by qRT-PCR; B: Representative western blot images of DNMT1 protein. H: mammary gland tissues from cows producing high quality milk; L: mammary gland tissues from cows producing low quality milk. The relative expression of DNMT1 was determined in two groups (n = 3 animals per group). QRT-PCR and western blotting assays were repeated three times for DNMT1 detection. C: Confocal microscopy images showing the localization of DNMT1 in dairy cow mammary gland tissue. H: mammary gland tissues from cows producing high quality milk; L: mammary gland tissues from cows producing low quality milk; (a) nuclear staining with propidium iodide, (b) DNMT1, and (c) merged image of (a) and (b). Mean optical densities of DNMT1 protein expression in mammary gland tissues from dairy cows with different milk qualities are shown in the graph. The data were analyzed with SPSS software. Values are means ± SD. DNMT1 is down-regulated in mammary gland tissues of cows producing high quality milk compared with those producing low quality milk. *P<0.05.

To determine the levels of maintenance methylation in dairy cow mammary gland tissues, we measured the levels of DNMT1 mRNA using qRT-PCR and found that DNMT1 was significantly down-regulated in the mammary gland tissues of cows producing H compared with that in cows producing L (Figure 2A, *P*<0.05). We also measured the DNMT1 protein levels using western blotting and immunohistochemistry techniques and found that the expression of DNMT1 was lower in mammary gland tissues from cows producing H compared with that from cows producing L as shown in Figure 2B and Figure 2C (*P*<0.05).

### DNMT1 is a direct target of miR-152

**Figure 3:**
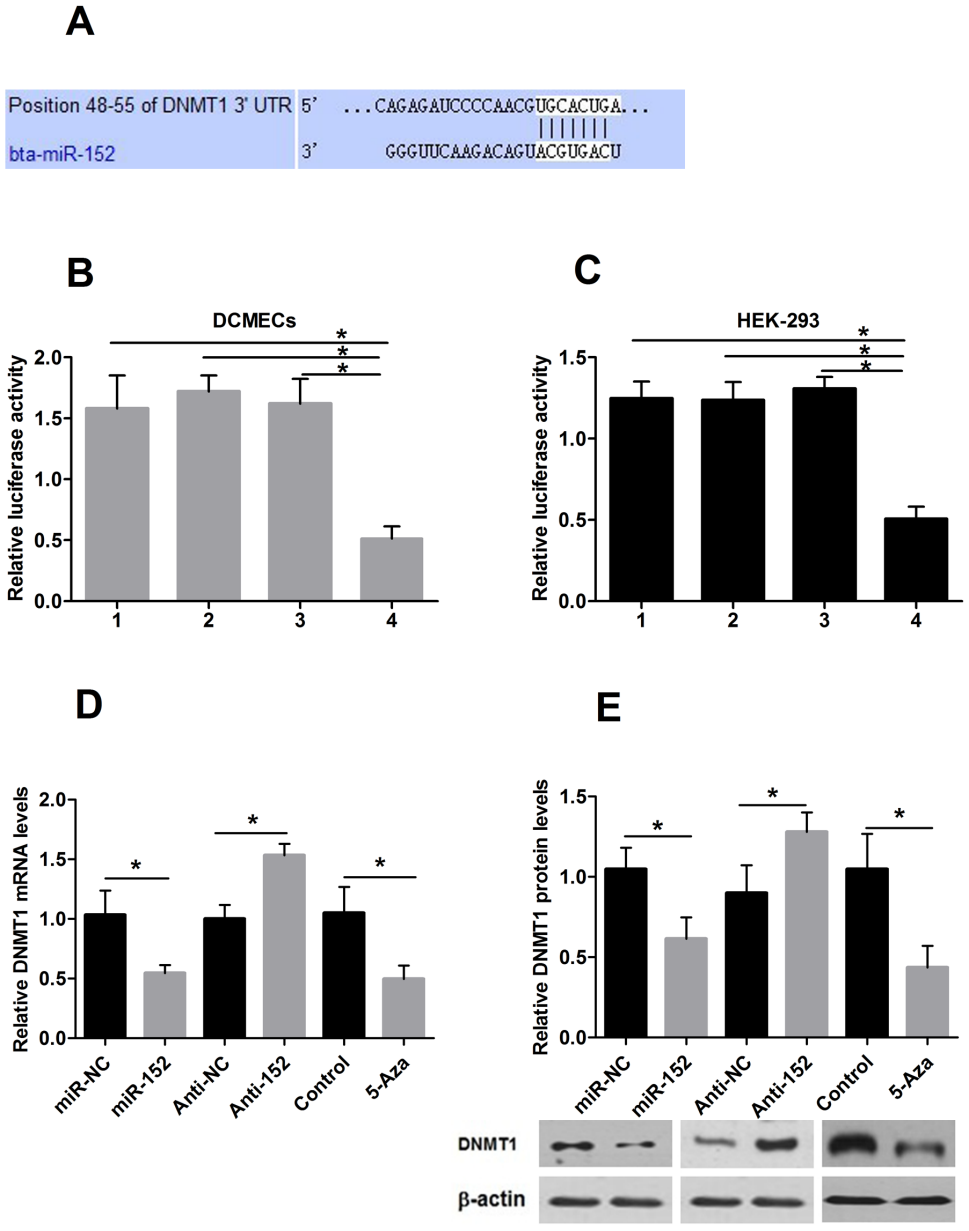
miR-152 regulates DNMT1 expression by binding its 3′ UTR. A: miR-152 binds to the 3′ UTR of DNMT1 in position 48–55. B, C: DNMT1 3′ UTR was inserted downstream of the luciferase pMIR-reporter vector. Luciferase activity was measured in DCMECs and HEK-293 cells. 1 phRL-TK and empty pMIR-REPORT vector cotransfection; 2 miR-152, phRL-TK, empty pMIR-REPORT vector cotransfection; 3 miR-NC, phRL-TK, DNMT1-pMIR-REPORT vector cotransfection and 4 miR-152, phRL-TK, DNMT1-pMIR-REPORT vector cotransfection. Luciferase activities of DNMT1-pMIR-REPORT are markedly decreased in cells transfected with miR-152 compared to those of reporter plasmids with miR-NC or empty vectors. D: *Dnmt1* mRNA expression after treatment with miR-152, Anti-152, 5-Aza or their respective controls in DCMECs. E: DNMT1 protein expression of DCMECs treated with miR-152, Anti-152, 5-Aza or their respective controls. MiR-152 targets DNMT1 and represses the expression of DNMT1 mRNA and protein. The mRNA and protein expression level of DNMT1 are significantly decreased after 48 h treatment with 5-Aza in DCMECs. Values are means ± SD, *P<0.05

The miR-152 targets predicted by computational algorithms [25] were obtained from TargetScan 4.0 and miRBase [26]. According to the prediction analysis, the key enzyme in DNA methylation, DNMT1, has a putative miR-152-binding site mapped to the 3′ UTR and was identified as one of the high-scoring candidate genes for miR-152 targeting, as shown in Figure 3A. To determine whether DNMT1 is regulated by miR-152 through direct binding to its 3′ UTR, the 3′ UTR was constructed and cloned downstream of the luciferase gene in a pMIR-Report vector (Ambion) and cotransfected with miR-152 or miR-NC in HEK-293 cells and in DCMECs. The results of the luciferase assay are shown in Figure 3B and Figure 3C. We observed a markedly lower luciferase activity in cells expressing the reporter with miR-152 than in those expressing the reporter with miR-NC (*P*<0.05). These results suggest that the gene for DNMT1 is a potential target for miR-152.

### MiR-152 inhibits DNMT1 expression

To test the hypothesis that miR-152 down-regulates DNMT1 in DCMECs, we transfected miR-152, Anti-152, or their respective controls into DCMECs. After 48 h, we measured the levels of the mRNA and protein expression for DNMT1. The results showed that the over-expression of miR-152 reduced DNMT1 expression at both the mRNA and protein levels in DCMECs. By contrast, the inhibition of miR-152 increased the DNMT1 expression. Subsequently, to confirm our results of an inverse correlation between miR-152 and DNMT1 levels, we found that demethylation treatment using 5-Aza blocked both de novo and maintenance DNMT pathways in DCMECs. The mRNA and protein expression levels of DNMT1 were significantly decreased after 48 h (Figure 3D, Figure 3E, *P*<0.05). However, the expression of DNMT1 in the group with over-expressed miR-152 was not significantly different than that in the group treated with 5-Aza (*P*>0.05).

### Under-expression of miR-152 increases GDM and DNMT activity

**Figure 4:**
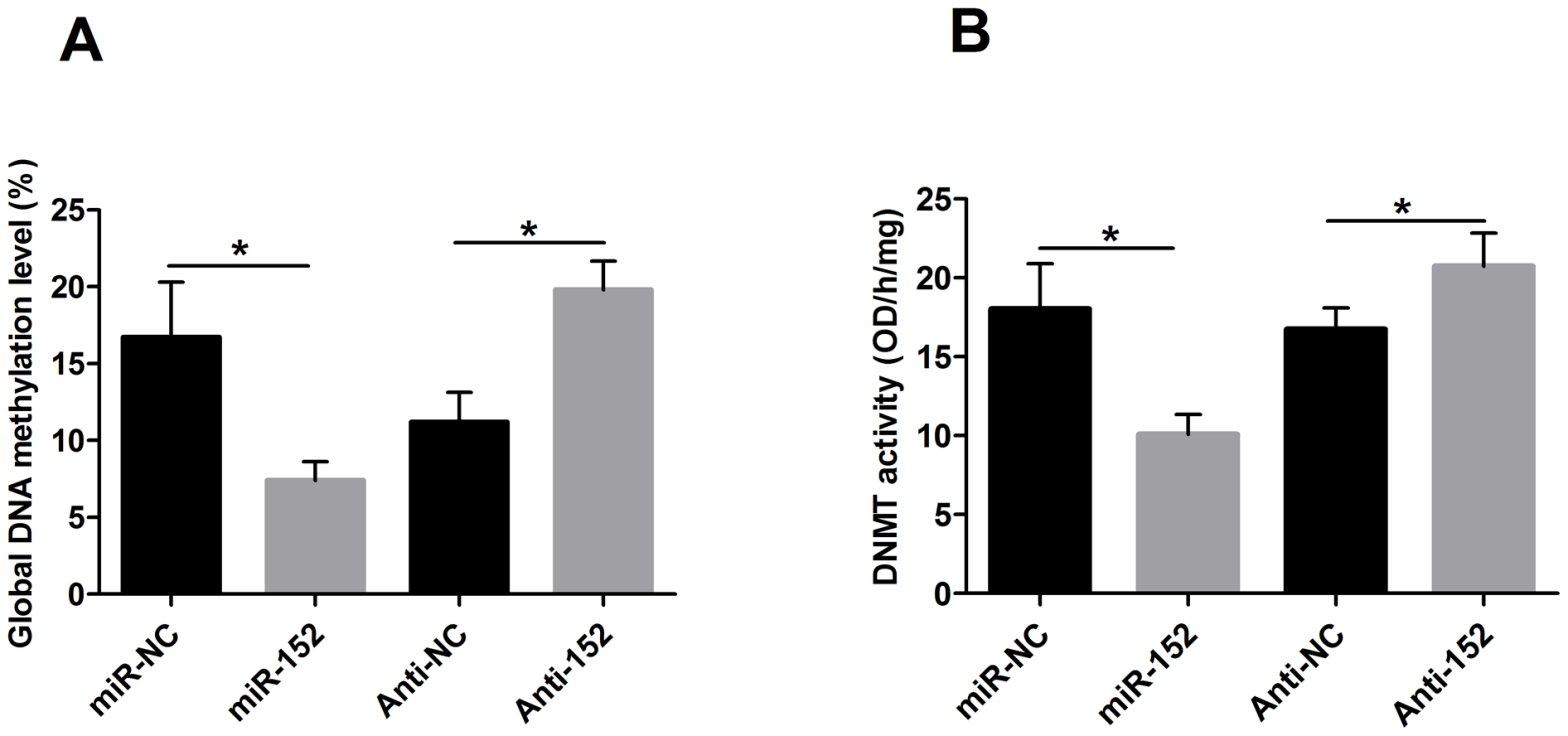
Under-expression of miR-152 induces aberrant DNA hypermethylation in DCMECs. A: GMD ratios of DCMECs after the transfection of miR-152, Anti-152, or their respective controls. B: DNMT activity of DCMECs after the transfection of miR-152, Anti-152, or their respective controls. GMD ratios and DNMT activity are markedly decreased in cells transfected with miR-152 compared to those values observed in miR-NC. Cells transfected with Anti-152 show the opposite results. Values are means ± SD, *P<0.05.

We also investigated whether the inhibition of miR-152 expression could lead to DNA hypermethylation and increase DNMT activity. GMD was measured with a 5-mC DNA ELISA Kit (ZYMO) in DCMECs after 48 h of transfection with miR-152, Anti-152, or their respective controls. We observed an increase of 7% in GDM for the DCMECs treated with Anti-152 compared with those treated with Anti-NC and a reduction of 9% in GDM for the DCMECs treated with miR-152 compared with those treated with miR-NC (Figure 4A, *P*<0.05). In addition, an EpiQuik DNA Methyltransferase assay kit (Epigentek) was used to detect the activity of DNMT according to the manufacturer's instructions. The activity of DNMT was significantly suppressed following over-expression of miR-152 compared to that following over-expression of miR-NC. The inhibition of miR-152 showed the opposite result (Figure 4B, *P*<0.05).

### MiR-152 increases the expression of AKT and PPARγ

**Figure 5:**
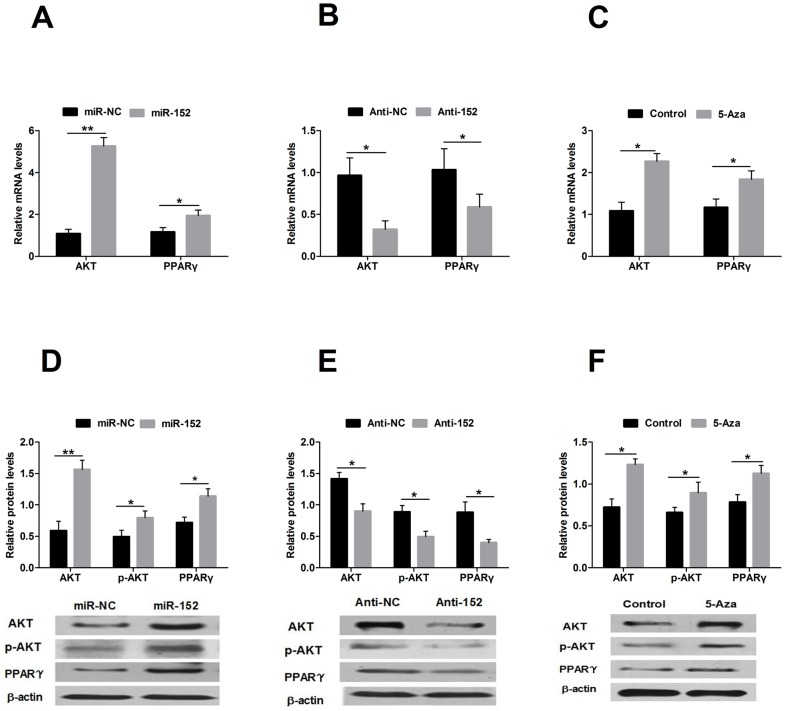
Expression of AKT and PPARγ during miR-152 over-expression and inhibition in DCMECs. A: mRNA levels of *Akt* and *Pparγ* following transfection of DCMECs with miR-152 and miR-NC; B: mRNA levels of *Akt* and *Pparγ* following transfection of DCMECs with Anti-152 and Anti-NC; C: mRNA levels of *Akt* and *Pparγ* following treatment with 5-Aza; D: Protein levels of AKT, p-AKT and PPARγ following transfection of DCMECs with miR-152 and miR-NC; E: Protein levels of AKT, p-AKT and PPARγ following transfection of DCMECs with Anti-152 and Anti-NC; F: Protein levels of AKT, p-AKT and PPARγ following treatment with 5-Aza. Expression levels are showed relative to the average expression of *β-Actin*. AKT and PPARγ are markedly increased in cells transfected with miR-152 compared with cells transfected with miR-NC. Cells transfected with Anti-152 show the opposite results. AKT and PPARγ are markedly higher in cells treated with 5-Aza than in control cells. Values are means ± SD, *P<0.05, **P<0.01.

To assess whether over-expressed miR-152 led to demethylation and induction of AKT and PPARγ in DCMECs through blocking DNMT1 expression in DCMECs, we first analyzed the expression of AKT and PPARγ. The expression levels of AKT and PPARγ mRNA were markedly increased (*P*<0.05, *P*<0.01, respectively) in the cells transfected with miR-152 compared with those levels in cells transfected with miR-NC (Figure 5A). By contrast, inhibition of miR-152 showed the opposite results (Figure 5B; *P*<0.05 and *P*<0.01, respectively). The protein levels for AKT, phospho-AKT (p-AKT) and PPARγ were also markedly increased in the miR-152 groups compared with those levels in the miR-NC groups (Figure 5D; *P*<0.05, *P*<0.01, *P*<0.05 respectively); the inhibition of miR-152 showed the opposite results (Figure 5E, *P*<0.05 for all comparisons).

To determine whether DNMT1 was associated with the reactivation of genes, DCMECs were treated with the demethylating agent 5-Aza. The results showed that AKT and PPARγ mRNA and protein expression levels were markedly higher in the cells treated with 5-Aza than in control cells, indicating that AKT and PPARγ were reactivated in DCMECs (Figure 5C and 5F, *P*<0.05 for all comparisons).

### MiR-152 promotes the proliferation of DCMECs

**Figure 6:**
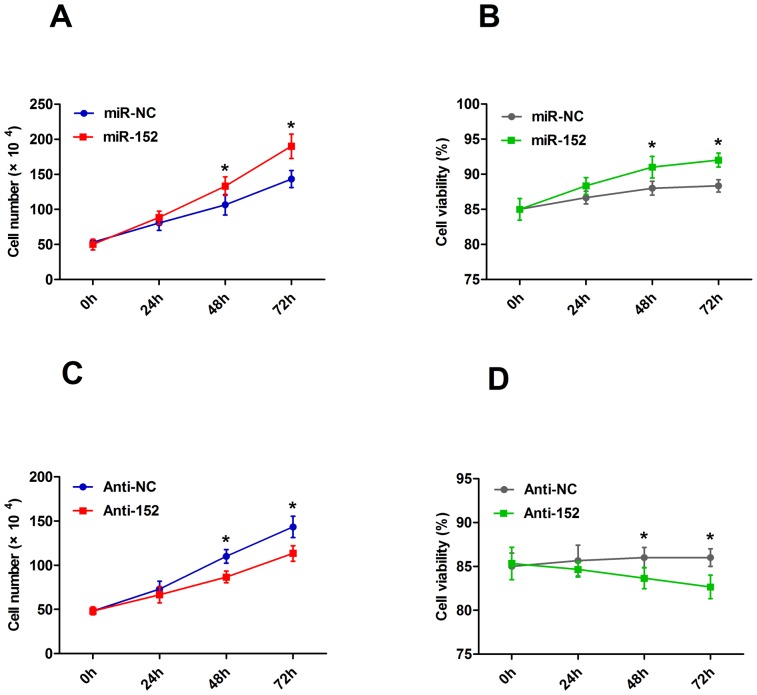
miR-152 promotes cell proliferation after transfection. Proliferation and viability of DCMECs were measured using CASY-TT after the transfection of miR-152, Anti-152, or their respective controls for 0 h, 24 h, 48 h, 72 h; A: Over-expression of miR-152 increases cell proliferation after 48 h and 72 h, whereas the inhibition of miR-152 show the opposite results. B: Over-expression of miR-152 increases cell viability, whereas the inhibition of miR-152 show the opposite results. Values are means ± SD, *P<0.05.

The CASY-TT Analyser System can determine the cell number and size distribution in the sample quickly and reliably. Moreover, the viability of the cells is measured directly, and the aggregation level of the cells is determined and automatically included in the calculation of the cell concentration. Here, cells were seeded on cell culture plates as described and harvested post-transfection. Cell viability was recorded as the percentage of viable cells in the total cells. The results showed that enhancing miR-152 caused an increase in total cell number as well as in cell viability in 48 h and 72 h (Figure 6A and 6B, *P*<0.05), whereas the inhibition of miR-152 showed the opposite results (Figure 6C, and 6D, *P*<0.05). These results suggested that miR-152 promoted cell proliferation and viability.

**Figure 7:**
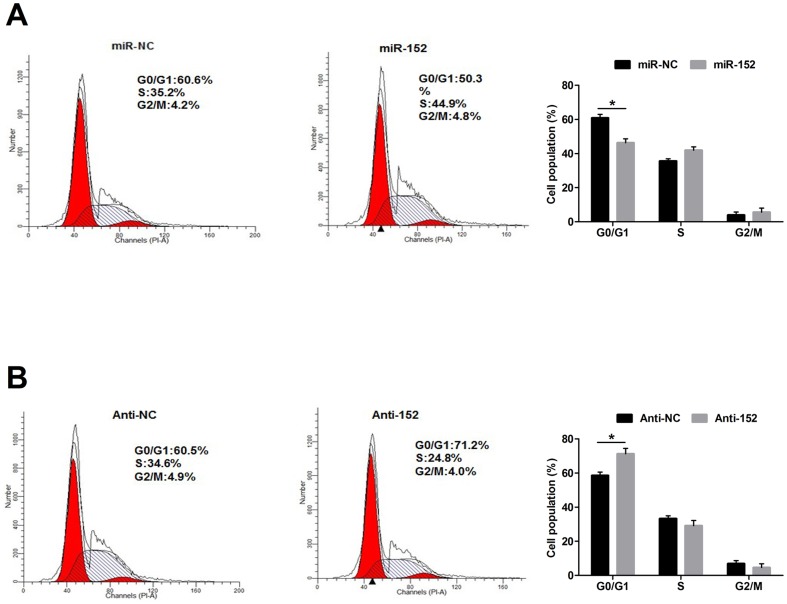
miR-152 regulates cell cycle after transfection. A: DCMECs were treated with miR-152 and miR-NC and stained with propidium iodide (PI) for flow cytometry. The population of DCMECs in G0/G1 phase following miR-152 treatment is decreased compared with that after miR-NC treatment; B: DCMECs were treated with Anti-152 and Anti-NC and stained with PI for flow cytometry. The population of DCMECs in G0/G1 phase after Anti-152 treatment is increased compared with that after Anti-NC treatment. Values are means ± SD, *P<0.05.

To dissect the mechanism of the proliferative effect of miR-152, we determined whether growth promotion was associated with specific cell cycle control. Flow cytometric analysis showed that, compared with DCMECs transfected with miR-NC, those transfected with miR-152 showed a significant decrease in the number of cells in the G0/G1 phase (Figure 7A, *P*<0.05). By contrast, inhibiting miR-152 resulted in the opposite effect (Figure 7B, *P*<0.05). These results indicated that the growth-promoting effect of miR-152 was due to a reduction in G0/G1 arrest.

### MiR-152 increases β-casein, triglyceride and lactose secretion of DCMECs

**Figure 8:**
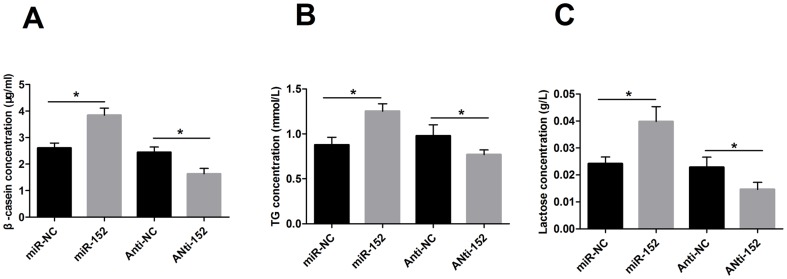
miR-152 influences secretion of β-casein, triglyceride and lactose in mammary epithelial cells. A: Secretion of β-casein after DCMECs were transfected with miR-152, Anti-152, or their respective controls; B: Secretion of triglyceride (TG) after DCMECs were transfected with miR-152, Anti-152, or their respective controls; C: Secretion of lactose after DCMECs were transfected with miR-152, Anti-152, or their respective controls. Secretion of β-casein, triglyceride and lactose is markedly increased in cells transfected with miR-152 compared with that in cells transfected with miR-NC. Cells transfected with Anti-152 show the opposite results. Values are means ± SD, * *P*<0.05.

To further investigate the influence of miR-152 on lactation, we determined the extracellular secretion of β-casein, triglyceride and lactose in DCMECs. As shown in Figure 8, over-expression of miR-152 markedly increased the levels of β-casein, triglyceride and lactose in cells compared with those levels in control groups. By contrast, the inhibition of miR-152 resulted in a significant decrease in these levels (*P*<0.05).

## Discussion

The small non-coding RNA gene products that are found in diverse organisms, including animals and plants, are known as miRNAs [27]. In mammals, miRNAs influence biological processes involved in development, tissue morphogenesis, tissue identity maintenance, cell growth, differentiation, apoptosis and metabolism. These miRNAs potentially regulate approximately 30% of all genes [6], [28]. Some miRNAs are reportedly related to breast cancer and mammary development, for example, miR-101a controls mammary gland development in the mouse by regulating cyclooxygenase-2 expression [6]. In a related study, Xu et al. found that a novel miR-148a/152-DNMT1 regulatory circuit might exist in breast cancer [20]. MiR-126-3p is specifically involved in mammary gland development in the mouse as well as in targeting and regulating the progesterone receptor, inhibiting proliferation of mammary epithelial cells in the mouse and secretion of β-casein [8]. MiR-15a regulates growth hormone receptor and changes the viability of cow mammary epithelial cells and the expression of casein [9]. These observations led us to postulate that post-transcriptional regulation by some miRNAs plays an important role in mammary development and lactation. However, the effect of miRNAs on mammary glands in cows is limited. In the present study, we used small RNA sequencing to investigate the levels of miRNA expression in mammary gland tissues from cows producing H and L and identified an miRNA, miR-152, the expression of which was increased in cows producing H compared with those producing L. This result was validated using qRT-PCR. Thus, we speculated that miR-152 might regulate mammary gland development through a mechanism different from those previously described.

Lactation, one of the most remarkable products of evolution, provides a unique model for studying basic biological processes, such as cell proliferation, differentiation, survival and death in mammary glands. The processes of lactation include the development of mammary gland tissue as well as the synthesis and secretion of milk. The main constituents in bovine milk are lactose, protein, and fat. It is well established that the synthesis and secretion of milk is under hormonal control [21]. Current knowledge of the molecular regulation of mammary development and lactation has largely been derived from the dissection of signaling networks in cell culture systems and from phenotypic characterization. Akt is activated in response to a number of hormones and growth factors that are involved in mammary gland development, and it has been implicated in regulating protein synthesis, lipid synthesis and glucose metabolism [12], [29]–[34]. PPARγ is a member of the nuclear receptor superfamily of transcription factors that can be activated by lipophilic ligands, and it is pivotal for maintaining the quality of milk and protecting nursing newborns by suppressing the production of inflammatory lipids in lactating mammary glands [35]. To understand the effects of miR-152 on the expression of the lactation signal transduction genes in the present study, we used western blotting and qRT-PCR analysis to observed alterations in DCMECs transfected with miR-152 mimics or inhibitors. The over-expression of miR-152 in DCMECs increased the mRNA and protein levels of AKT and PPARγ compared with those levels in control groups, whereas the inhibition of miR-152 showed the opposite results. In addition, the levels of β-casein, triglyceride and lactose were markedly increased by miR-152 over-expression and decreased by inhibition of miR-152 compared to those levels in control groups. These results indicated that miR-152 can enhance the PI3K/Akt signaling pathway and increase PPARγ and the secretion of β-casein, triglyceride and lactose.

DNA methylation is the best-studied epigenetic mechanism. In mammals, DNA methylation is a post-replication modification that is predominantly found in the cytosines of the dinucleotide sequence CpG. DNMT1 alone is required to maintain a large degree of global and CpG island methylation in human cancer cells. Most CpG islands are associated with genes, and all may contain promoters. Transcriptional silencing by CpG island methylation is a prevalent mechanism of tumor-suppressor genes in cancers [14], [36], [37]. It has been reported that during the neurogenic period, DNA methylation inhibits not only astroglial marker genes but also genes that are essential for JAK-STAT signaling [38]. It was demonstrated that the methylation status of the STAT5 promoter region acutely affects α-casein expression in bovine mammary gland tissue [12], [13]. Informed by these results, we postulated that the methylation status of the lactation signal transduction gene promoters can affect PI3K/Akt signaling and PPARγ, which in turn influence mammary gland development and lactation. An increasing amount of evidence indicates that miRNAs are involved in aberrant DNA hypermethylation through their regulation of DNMTs. It was reported that miR-152 can target DNMT1 and induce aberrant DNA methylation in HBV-related hepatocellular carcinoma [15] and that miR-152 targets DNMT1 in Nis-transformed cells via a feedback mechanism [37], [39]. The miR-29 family reverts aberrant methylation in lung cancer by targeting DNMT3a and DNMT3b [19]. In addition, miR-152 and miR-185 were shown to be involved in cisplatin-resistant ovarian cancer in vitro and in vivo through their direct targeting of DNMT1 [40]. In the present study, we showed that miR-152 exerted its effects by specifically targeting DNMT1 in cow mammary glands. We provided evidence using luciferase activity assays to suggest that DNMT1 is a potential target of miR-152, indicating that miR-152 is involved in the regulation of DNMT1. Furthermore, we confirmed that the levels of miR-152 are inversely correlated with those of DNMT1 in cow mammary gland tissues. The over-expression of miR-152 repressed the expression of DNMT1 at both the mRNA and protein levels in DCMECs. We also provided insights regarding the involvement of miR-152 in the control of DNA methylation mediated through the targeting of DNMT1 in DCMECs by measuring GDM and the activity of DNMT. The over-expression of miR-152 led to a decrease in GDM and in the activity of DNMT. The inhibition of DNMT1 by the over-expression of miR-152 significantly enhanced demethylation, which in turn up-regulated the expression of AKT and PPARγ. The expression of AKT and PPARγ was consistent with the effect of treatment with the DNMT1 inhibitor 5-Aza. 5-Aza inhibited the expression of DNMT1, which in turn enhanced the expression of AKT and PPARγ. These results showed that miR-152 down-regulated DNMT1 to inhibit GDM and the activity of DNMT, leading to a higher expression of AKT and PPARγ.

It has been reported that the ability of mammary glands in ruminants to produce milk is determined by the number and activity of secreting cells. An increase in milk yield is associated with increasing mammary epithelial cell activity, survival, and proliferation in lactating dairy cows [41]. Our results showed that the over-expression of miR-152 increased the viability of DCMECs, while the inhibition of miR-152 decreased the viability of DCMECs, suggesting that miR-152 may regulate cell viability. Moreover, miR-152 was involved in the regulation of cell cycle progression. Our results demonstrated that the transfection of miR-152 decreased the population of DCMECs in the G1 phase, indicating that miR-152 accelerated cell cycle progression. Here we suggest that miR-152 functions as a novel regulator to promote proliferation in DCMECs proliferation. However, the role of miR-152 in cell cycle progression warrants further study.

## Conclusion

In summary, our results revealed that miR-152 was specifically involved in mammalian mammary gland development and lactation. Through down-regulating the expression of DNMT1, miR-152 reduced GDM and the activity of DNMT to reactivate the lactation signal transduction genes *Akt* and *Pparγ*. In addition, miR-152 significantly altered the viability of the DCMECs as well as the secretion of β-casein, triglyceride and lactose. These results imply that miRNAs cannot be overlooked as a class of molecules regulating biological functions and mammary gland development.

## Supporting Information

Figure S1 **Cultured dairy cow mammary gland epithelial cells (DCMECs).** A: DCMECs were observed under light microscope (200×); B: Cytokeratin-18 staining of mammary epithelial cells (400×). The nucleus is stained red with propidium iodide (PI), and cytokeratin-18 is labeled with green fluorescence using FITC.
(TIF)
