# Expression of concern: MicroRNA-152 regulates DNA methyltransferase 1 and is involved in the development and lactation of Mammary Glands in Dairy Cows

**DOI:** 10.1371/journal.pone.0234680

**Published:** 2020-06-29

**Authors:** The PLOS ONE Editors

After publication of this article [1], concerns were raised about similarities between the following figure panels:

- Left (miR-NC/miR-152) and middle (Anti-NC/Anti-152) β-actin panels in Fig 3E.
- AKT panels Fig 5E and 5F (when flipped horizontally).

**Fig 3:**
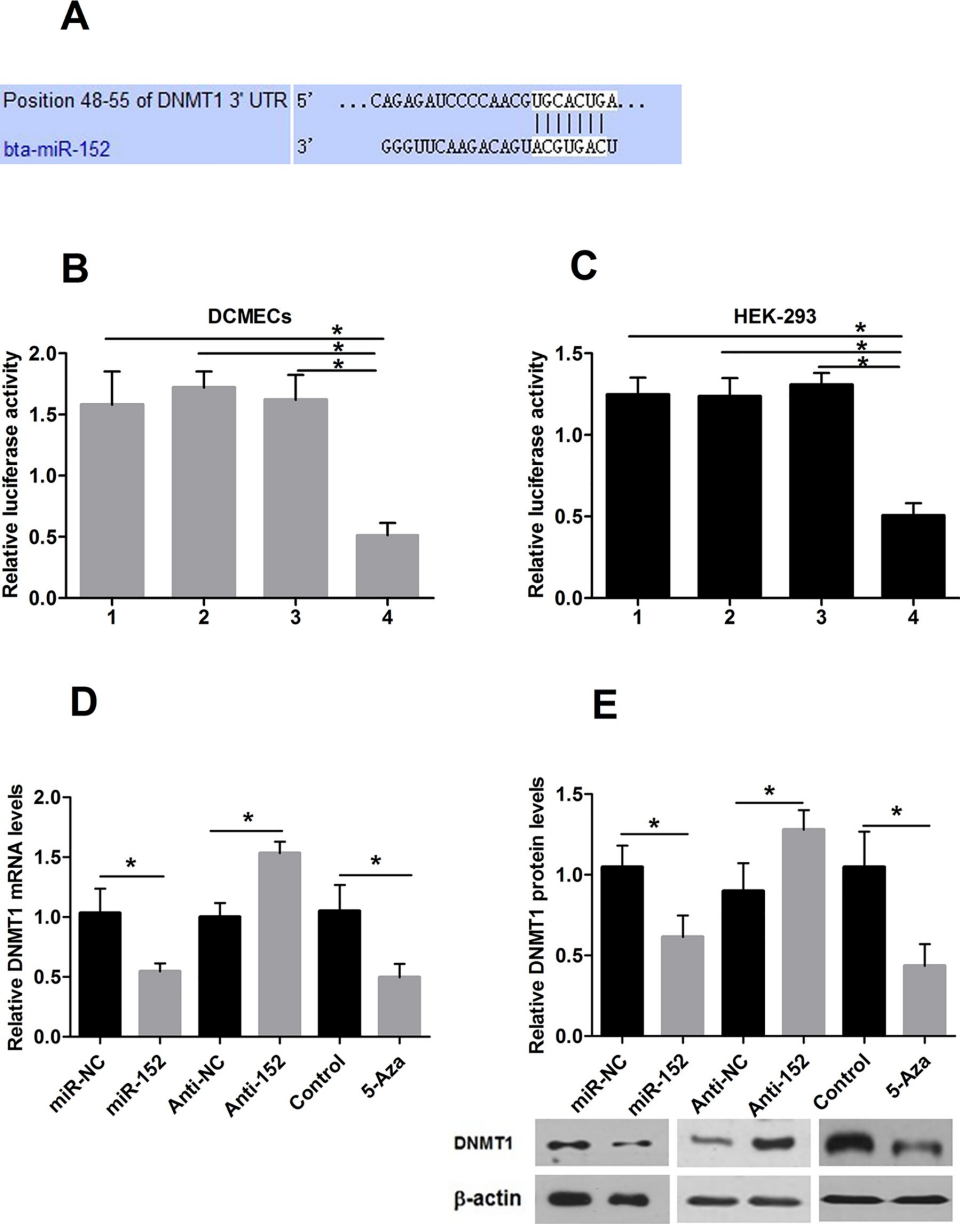
miR-152 regulates DNMT1 expression by binding its 3′ UTR. A: miR-152 binds to the 3′ UTR of DNMT1 in position 48–55. B, C: DNMT1 3′ UTR was inserted downstream of the luciferase pMIR-reporter vector. Luciferase activity was measured in DCMECs and HEK-293 cells. 1 phRL-TK and empty pMIR-REPORT vector cotransfection; 2 miR-152, phRL-TK, empty pMIR-REPORT vector cotransfection; 3 miR-NC, phRL-TK, DNMT1-pMIR-REPORT vector cotransfection and 4 miR-152, phRL-TK, DNMT1-pMIR-REPORT vector cotransfection. Luciferase activities of DNMT1-pMIR-REPORT are markedly decreased in cells transfected with miR-152 compared to those of reporter plasmids with miR-NC or empty vectors. D: *Dnmt1* mRNA expression after treatment with miR-152, Anti-152, 5-Aza or their respective controls in DCMECs. E: DNMT1 protein expression of DCMECs treated with miR-152, Anti-152, 5-Aza or their respective controls. MiR-152 targets DNMT1 and represses the expression of DNMT1 mRNA and protein. The mRNA and protein expression level of DNMT1 are significantly decreased after 48 h treatment with 5-Aza in DCMECs. Values are means ± SD, *P<0.05.

**Fig 5:**
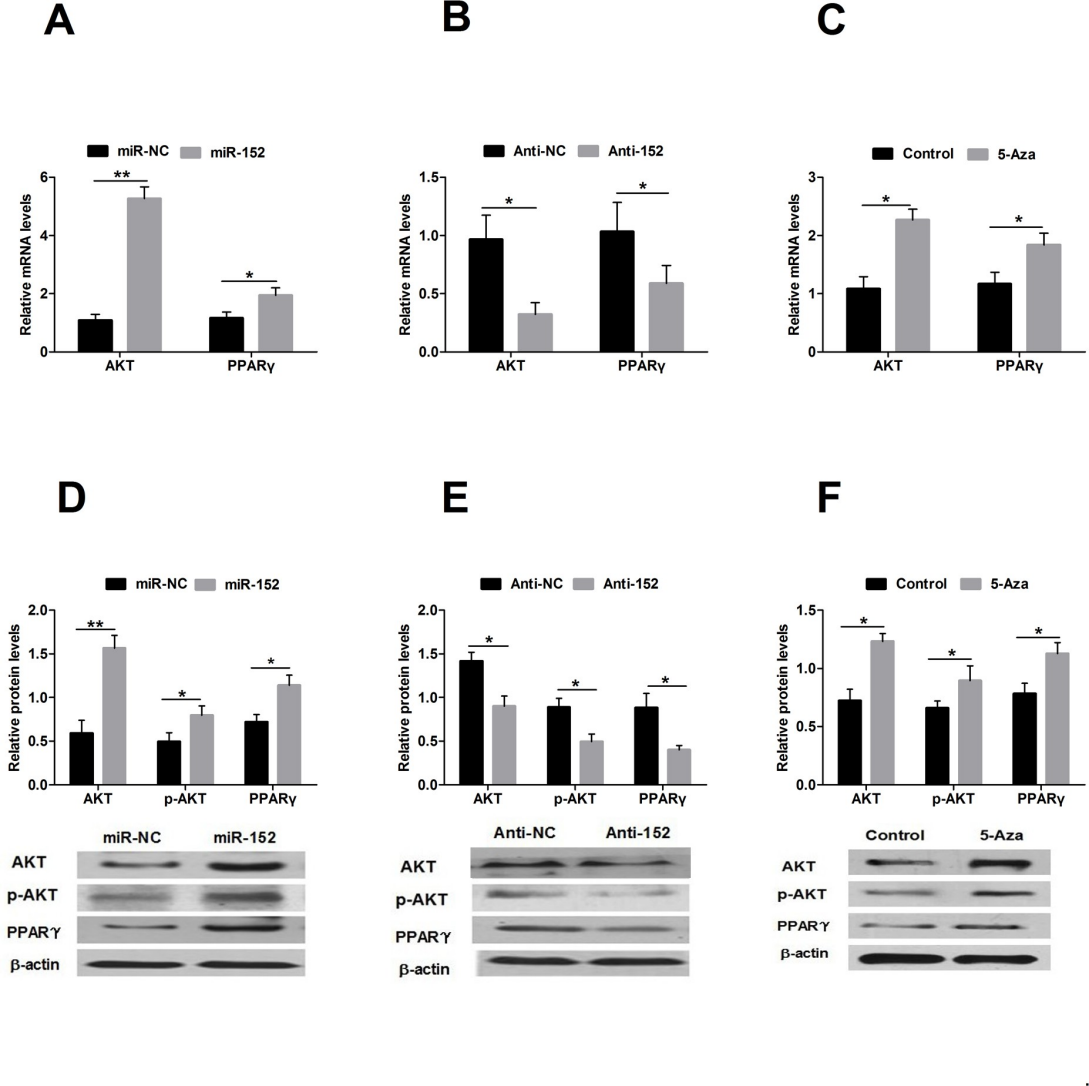
Expression of AKT and PPARγ during miR-152 over-expression and inhibition in DCMECs. A: mRNA levels of *Akt* and *Pparγ* following transfection of DCMECs with miR-152 and miR-NC; B: mRNA levels of *Akt* and *Pparγ* following transfection of DCMECs with Anti-152 and Anti-NC; C: mRNA levels of *Akt* and *Pparγ* following treatment with 5-Aza; D: Protein levels of AKT, p-AKT and PPARγ following transfection of DCMECs with miR-152 and miR-NC; E: Protein levels of AKT, p-AKT and PPARγ following transfection of DCMECs with Anti-152 and Anti-NC; F: Protein levels of AKT, p-AKT and PPARγ following treatment with 5-Aza. Expression levels are showed relative to the average expression of *β-Actin*. AKT and PPARγ are markedly increased in cells transfected with miR-152 compared with cells transfected with miR-NC. Cells transfected with Anti-152 show the opposite results. AKT and PPARγ are markedly higher in cells treated with 5-Aza than in control cells. Values are means ± SD, *P<0.05, **P<0.01.

The authors note that these duplications arose due to errors in assembling Fig 3E (left β-actin panel) and 5E. Updated figures are provided here in which the left β-actin panel of Fig 3 and the AKT panel of Fig 5E were replaced with the correct images from the original experiments. Image data provided in support of the four panels in question are in S1–S4 Files; the original image data to support other panels in Figs 3E, 5E and 5F are no longer available. Note that the images are compressed vertically in the updated figures as compared to the supporting image files.

The authors indicated that the quantification results shown in Figs 3E, 5E and 5F were obtained using the correct blots and are not affected by the figure errors. The raw quantitative data supporting these graphs are no longer available.

The underlying data supporting all other results reported in the article are no longer available.

The *PLOS ONE* Editors issue this Expression of Concern in light of the unavailability of data to support the results reported in the article.

The authors apologize for the errors in the published figures.

## Supporting information

S1 File Correct control blot for the left panel of the original Fig 3E. (TIF)

S2 File Correct control blot for the middle panel of the original Fig 3E. (TIF)

S3 File Correct AKT control blot for the original Fig 5E. (TIF)

S4 File Correct AKT control blot for the original Fig 5F. (TIF)
