# Supplementary figures and images for: MicroRNA-152 Regulates DNA Methyltransferase 1 and Is Involved in the Development and Lactation of Mammary Glands in Dairy Cows
Source: PLoS One. 2014 Jul 2;9(7):e101358. doi: 10.1371/journal.pone.0101358 (PMC4079547; doi:10.1371/journal.pone.0101358)

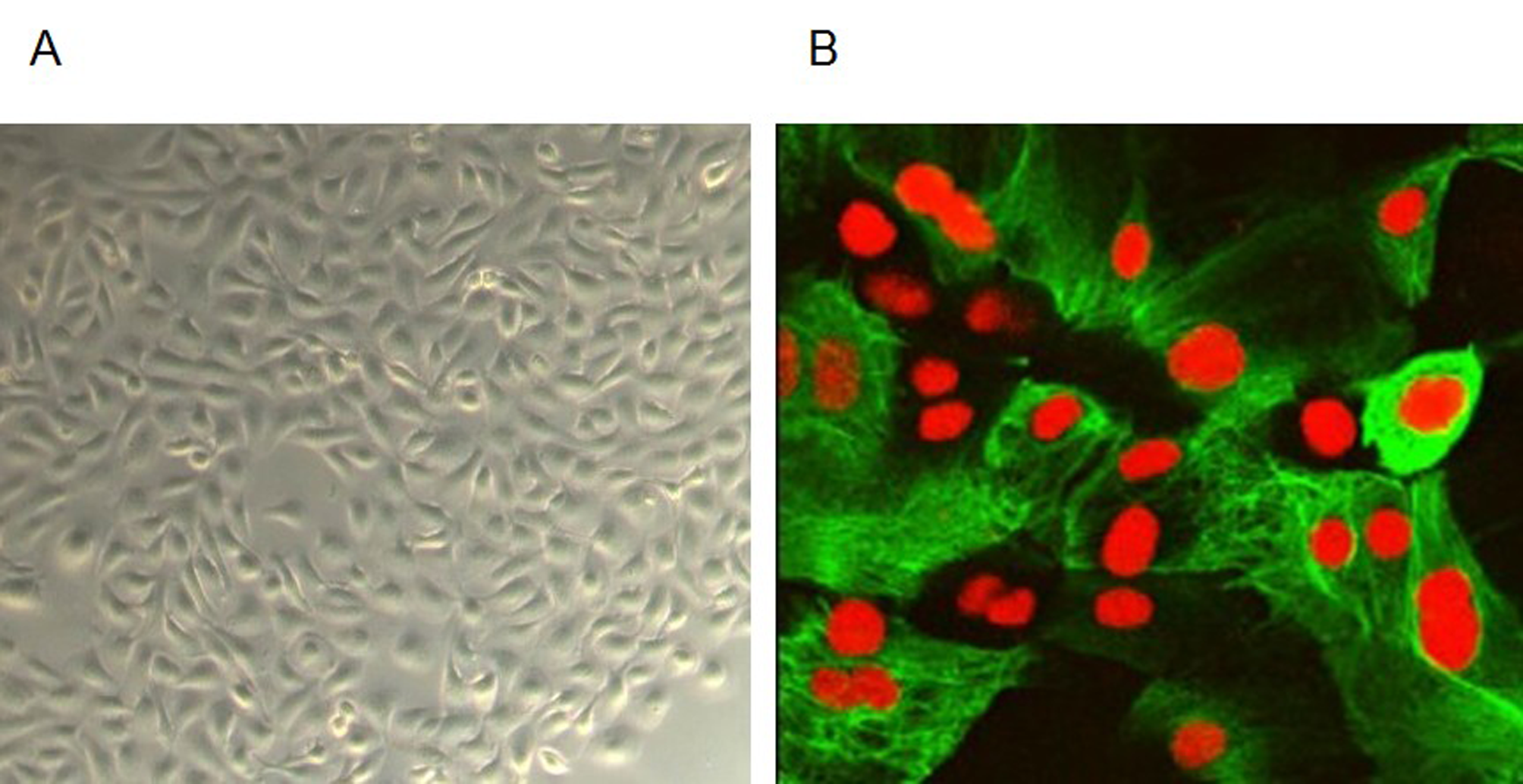

Supplement: Figure S1 — Cultured dairy cow mammary gland epithelial cells (DCMECs). A: DCMECs were observed under light microscope (200×); B: Cytokeratin-18 staining of mammary epithelial cells (400×). The nucleus is stained red with propidium iodide (PI), and cytokeratin-18 is labeled with green fluorescence using FITC. (TIF) [file pone.0101358.s001.tif]
