# Supplementary figures and images for: Expression of concern: MicroRNA-152 regulates DNA methyltransferase 1 and is involved in the development and lactation of Mammary Glands in Dairy Cows
Source: PLoS One. 2020 Jun 29;15(6):e0234680. doi: 10.1371/journal.pone.0234680 (PMC7323983; doi:10.1371/journal.pone.0234680)

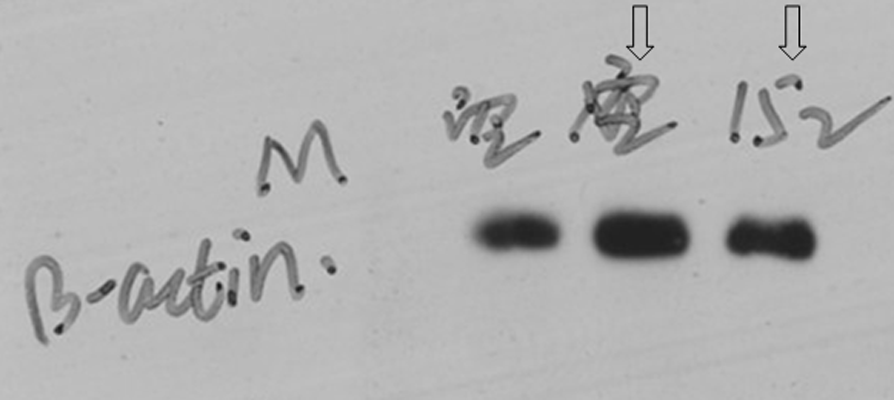

Supplement: S1 File — (TIF) [file pone.0234680.s001.tif]

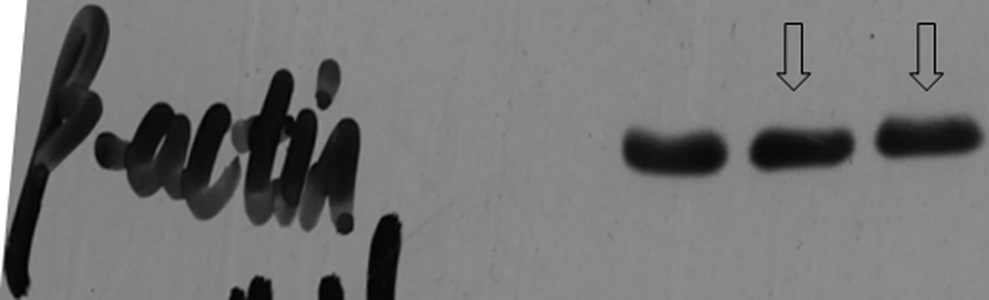

Supplement: S2 File — (TIF) [file pone.0234680.s002.tif]

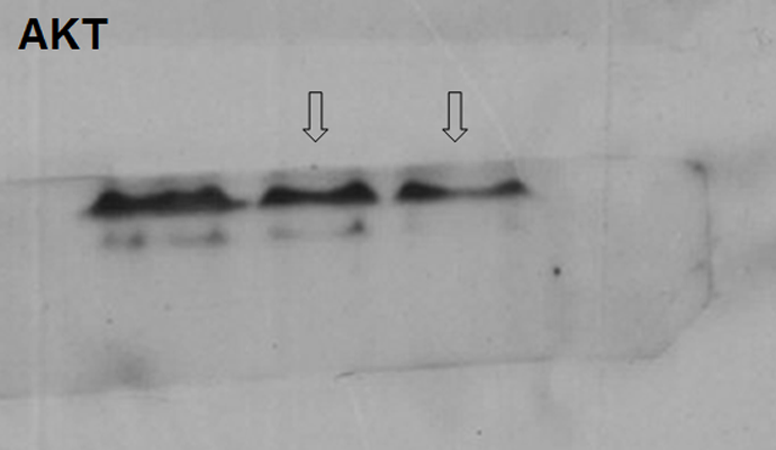

Supplement: S3 File — (TIF) [file pone.0234680.s003.tif]

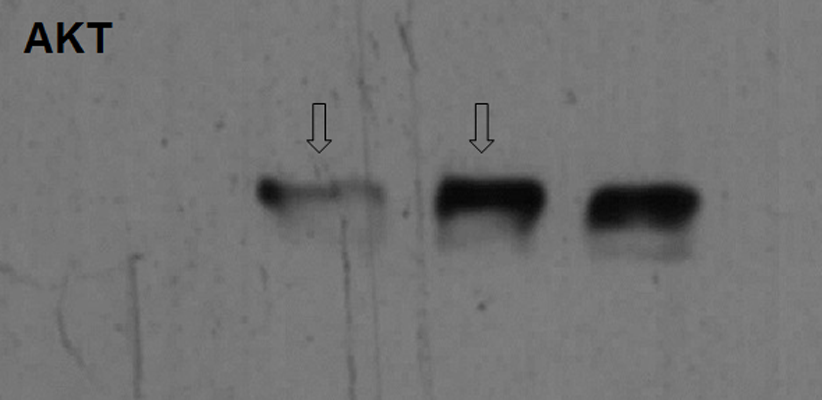

Supplement: S4 File — (TIF) [file pone.0234680.s004.tif]
